# Premalignant Lesions in the Kidney

**DOI:** 10.1100/tsw.2001.321

**Published:** 2001-12-07

**Authors:** Ziva Kirkali, Kutsal Yorukoglu

**Affiliations:** ^1^ Department of Urology, Eylul University, School of Medicine, 35340 Inciralti, Izmir, Turkey; ^2^ 2Department of Pathology, Dokuz Eylul University, School of Medicine, 35340 Inciralti, Izmir, Turkey

**Keywords:** renal cell carcinoma, dysplasia, precursor lesions

## Abstract

Renal cell carcinoma (RCC) is the most malignant urologic disease. Different lesions, such as dysplasia in the tubules adjacent to RCC, atypical hyperplasia in the cyst epithelium of von Hippel-Lindau syndrome, and adenoma have been described for a number of years as possible premalignant changes or precursor lesions of RCC. In two recent papers, kidneys adjacent to RCC or removed from other causes were analyzed, and dysplastic lesions were identified and defined in detail. Currently renal intraepithelial neoplasia (RIN) is the proposed term for classification. The criteria for a lesion to be defined as premalignant are (1) morphological similarity; (2) spatial association; (3) development of microinvasive carcinoma; (4) higher frequency, severity, and extent then invasive carcinoma; (5) progression to invasive cancer; and (6) similar genetic alterations. RIN resembles the neoplastic cells of RCC. There is spatial association. Progression to invasive carcinoma is described in experimental cancer models, and in some human renal tumors. Similar molecular alterations are found in some putative premalignant changes. The treatment for RCC is radical or partial nephrectomy. Preneoplastic lesions may remain in the renal remnant in patients treated by partial nephrectomy and may be the source of local recurrences. RIN seems to be a biologic precursor of some RCCs and warrants further investigation. Interpretation and reporting of these lesions would reveal important resources for the biological nature and clinical significance. The management of RIN diagnosed in a renal biopsy and partial nephrectomy needs to be answered.

## INTRODUCTION

A number of organs, such as the uterine cervix[1], urothelium[2], respiratory epithelium[3], and gastrointestinal tract[4] have well-recognized premalignant lesions. In contrast, premalignant lesions of the kidney are poorly described. This is due to the lack of a practical screening method for kidney tumors. And, the kidney can be examined pathologically only in tru-cut biopsies of glomerular diseases and in nephrectomy specimens of neoplastic lesions or end-stage renal failure.

To be defined as premalignant (dysplasia), a lesion should meet a number of criteria[5]. These are the following:

1. They should have morphologic features similar to invasive carcinoma.
2. There should be a spatial association between the lesion and invasive carcinoma.
3. Foci of microinvasive cancer should be seen to arise from the putative premalignant change, and progression to invasive cancer should be described in some conditions.
4. The frequency, severity, and extent of the lesion should occur to a greater degree than invasive cancer.
5. Genetic alterations, if any could be shown in these lesions, would be the definite evidence.

## Renal Dysplasia Morphology

Renal intratubular neoplasia is defined as epithelial crowding by large nuclei, an increased nucleus/cytoplasm ratio, hyperchromasia, clumped chromatin, and irregularity of the nuclear membrane[6,7] (Figs. 1,2,3,4). The morphologic features have been defined in both experimental and clinical studies[6,7,8,9]. The cells in preneoplastic lesions associated with renal cell carcinoma (RCC) resemble the neoplastic cells. Dysplastic lesions were described in association of cystic RCC[10].

Dysplasia grade seems to correlate with the presence of carcinoma. Dysplastic changes in the tubules of the normal kidneys were not as severe as those identified in carcinoma cases[6].

## Renal Dysplasia Spatial Association with Carcinoma

In the detailed two series examining kidney parenchyma for the dysplastic changes, preneoplastic lesions were identified mainly in the cortex, similar to RCC. Some of the dysplastic changes were noted in the medullary region[6,7]. This is also true for collecting duct carcinoma[11,12]. The presence of dysplastic features in the epithelium of collecting ducts neighboring a tumor may be considered as evidence that the tumor originated in the epithelium of the collecting ducts. The presence of epithelial dysplasia in collecting ducts remote from the tumor is a definite evidence of a premalignant condition in the collecting ducts rather than abnormalities in nearby ducts.

## Renal Dysplasia Progression

One of the definitive requirements for a lesion to be considered premalignant is the observation of its progression into invasive carcinoma on serial biopsy. Microinvasive neoplasms from proximal tubule dysplasias and oncocytomas from preneoplastic lesions were shown to develop in experimental models[8,13,14]. It is not easy to serially biopsy the tubular system due to the three-dimensional characteristics and to demonstrate histologically the progression of dysplasia into invasive carcinoma. Despite this limitation, it appears that dysplasia is a premalignant lesion in the human kidney. The suggestion that dysplasia may be a tumor-associated condition, but not premalignant, does not reflect the frequent observation of dysplasia in organs without invasive carcinoma.

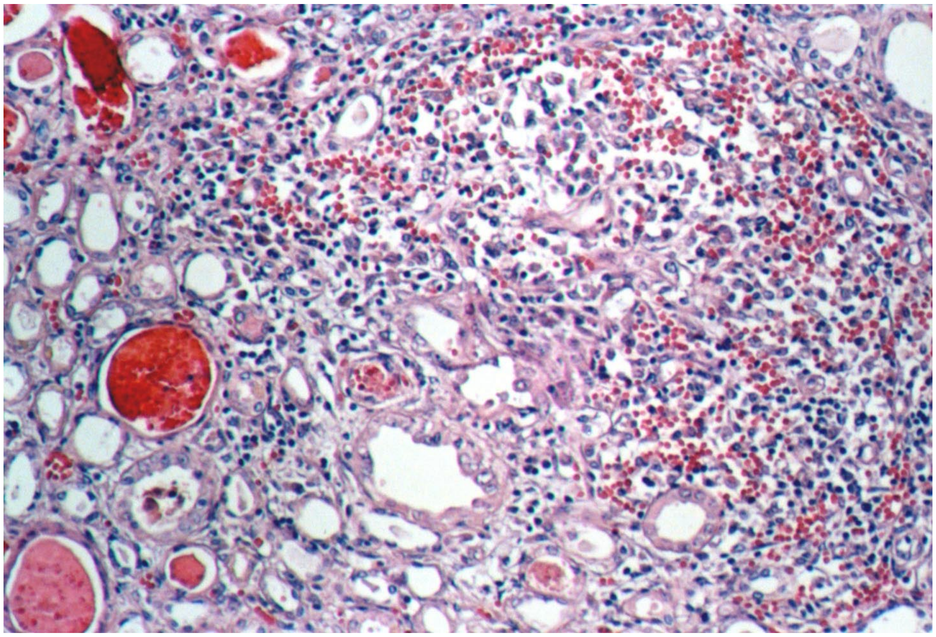

**FIGURE 1.** Dysplastic changes in the tubules adjacent to RCC. Nuclear size is increased. Nuclei are vesicular and pleomorphic. Interstitial fibrosis, erythrocyte extravasation, and lymphoplasmacytic infiltration are noted.

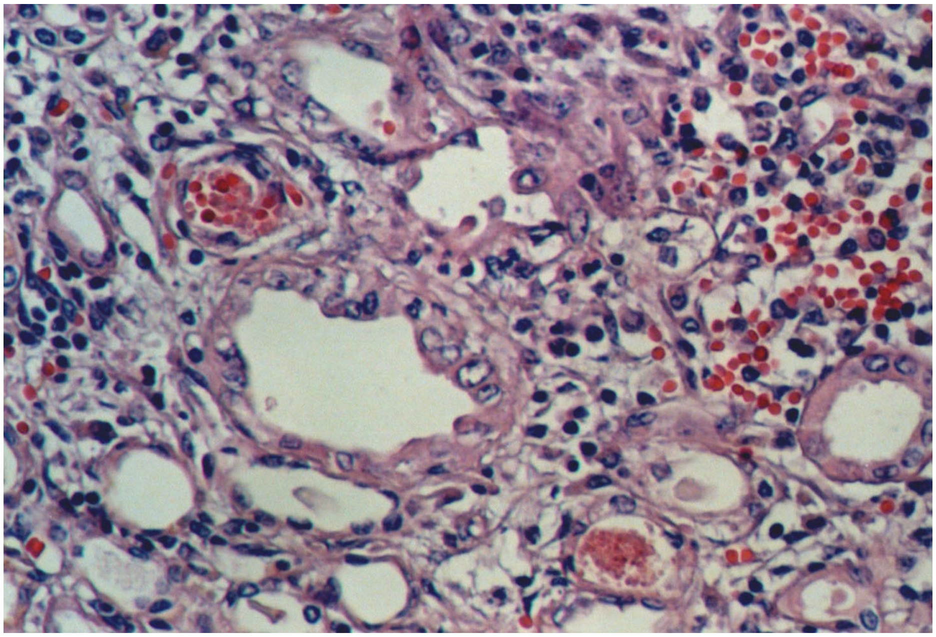

**FIGURE 2.** In high-power view, nuclear atypia and pleomorphism are clearly seen.

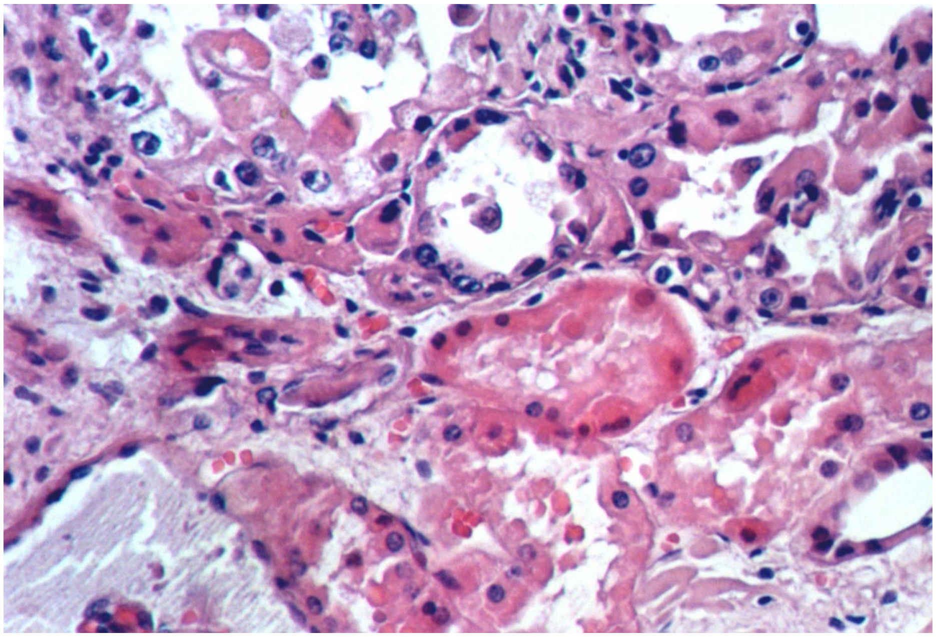

**FIGURE 3.** Dysplastic tubules are at the upper half adjacent to normal tubules placed at the lower half of the figure. The nuclear size and chromatin staining is increased, and the nucleoli are prominent in the dysplastic tubules. There is some nuclear crowding and multilayering.

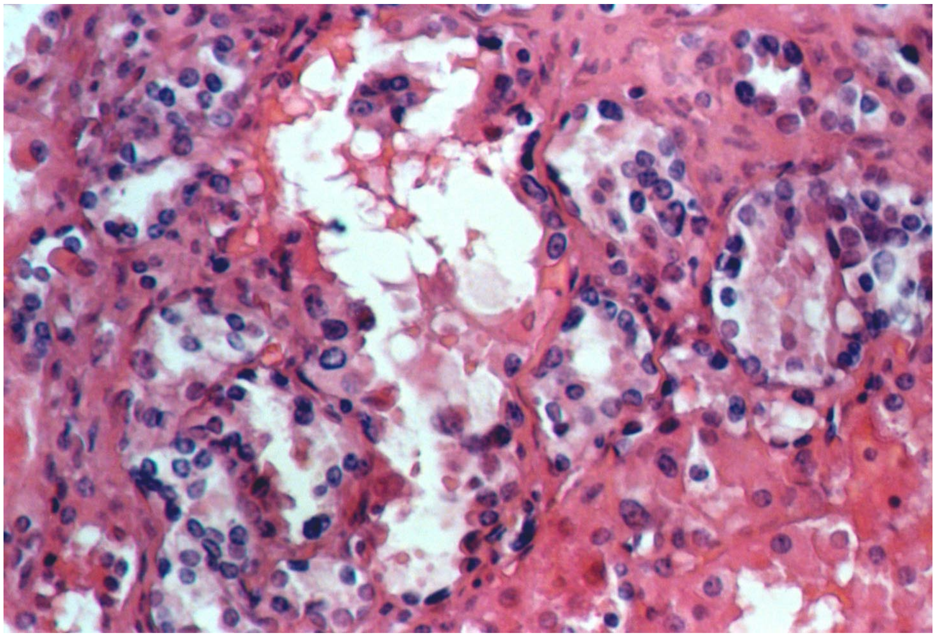

**FIGURE 4.** Focal dysplastic changes in the tubules in an autopsy case with entirely normal kidneys.

## Renal Dysplasia Incidence

Another requirement for a lesion to be considered premalignant is that its incidence be higher in organs with carcinoma than in those without. In two series with 110 and 62 RCC cases, the incidence of dysplasia was 28 and 23%, respectively[6,7]. In these series, the incidence in the control group was 0 and 17.4%, respectively. Epithelial cortical lesions (papillary adenoma) were found in 21% of 400 autopsy cases[15], and the incidence has been found variously to be between 4 and 37% in the other autopsy patients[16,17]. But the kidneys with RCC or oncocytoma are more likely to contain papillary adenoma than normal kidney[18]. This may be the result of some local factors promoting the development of adenomas.

The incidence of dysplasia in organs without carcinoma is considerably lower than the incidence in kidneys with cancer, but it is still quite high. The disparity in the reported incidence may be related in part to the differences in the specimen handling[19].

## Renal Dysplasia Genetic Alterations

Cytogenetic classification is presumed to be a more appropriate strategy than histopathological one in RCC[20]. The morphological subtypes of RCC are shown to exhibit different cytogenetic anomalies suggesting different molecular alteration pathways in the development of these variants[21].

Hereditary inactivation of tuberous sclerosis 2 tumor suppressor gene predisposes the Eker rat to develop RCC, and overexpression of transforming growth factor $\alpha$ (TGF-$\alpha$) was found to occur both in adenomas and dysplasias[22]. Epidermal growth factor (EGF) is the cognate receptor of TGF-$\alpha$, and dysplastic lesions in RCC cases showed an increased incidence of expression of EGF-receptor compared with the nondysplastic lesions[7].

The tubules adjacent to RCC with no evidence of intraepithelial dysplasia showed focal p53 immunoexpression[23] (Fig. 5). This mutant p53 expression provides supportive evidence that previously described intraepithelial dysplasia in kidneys adjacent to RCC are most likely to be precursor lesions. Aberrant expression of p53 in areas without evidence of intraepithelial dysplasia may suggest that neoplastic transformation manifested by p53 mutation in kidney tubules may be seen before the development of the morphologic features of dysplasia and malignancy. The presence of such markers in the dysplastic epithelium, however, may be further proof that they present true neoplastic transformation in the tubular epithelium rather than a reactive process.

Some atypical cysts in von Hippel-Lindau syndrome show a loss of the wild type VHL allele as in RCC[23].

Cytogenetic data of papillary adenomas and papillary RCC show similar findings (trisomies of chromosome 7 and 17 with loss of the Y chromosome in males)[24,25]. The presence of similar molecular alterations in these lesions and RCC suggests them to be true preneoplastic lesions. It was shown that dysplastic tubular epithelium is more intensely stained for proliferating cell nuclear antigen (PCNA) than normal epithelium and carcinoma cells[6] (Figs. 6,7,8). Also, chromosomal abnormalities that are found in RCC were found in the noncancerous renal tissue[26] and in normal kidneys[27].

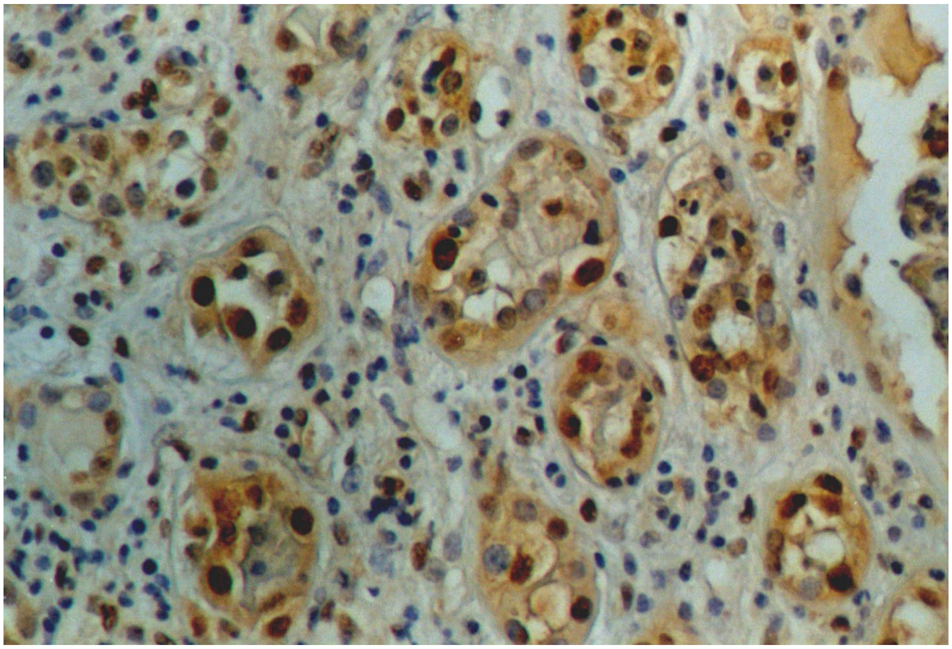

**FIGURE 5.** p53 immunoexpression in normal tubules adjacent to RCC.

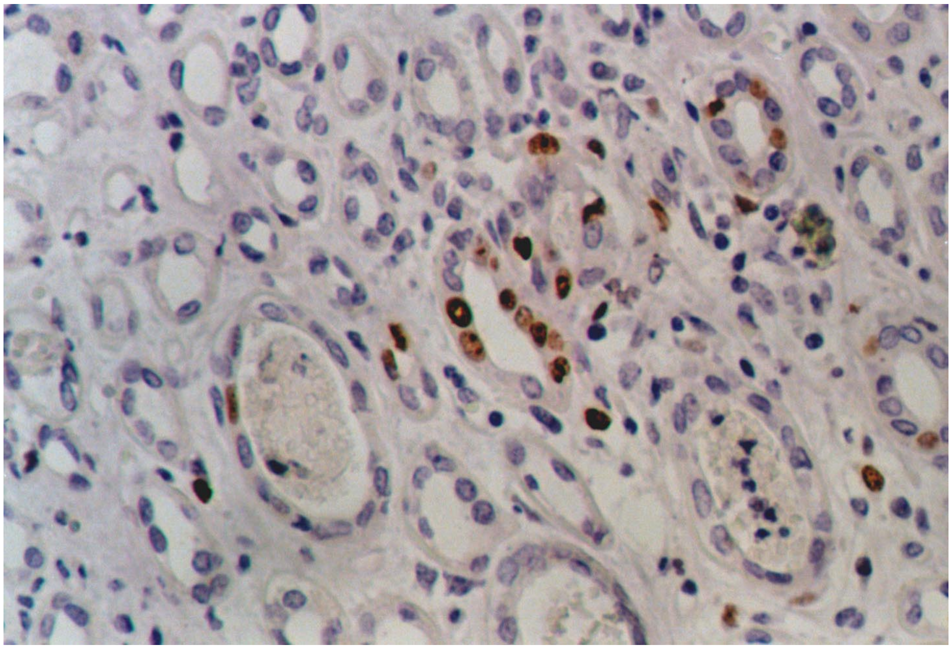

**FIGURE 6.** Proliferating cell nuclear antigen (PCNA) immunostaining in dysplastic tubular epithelium cells.

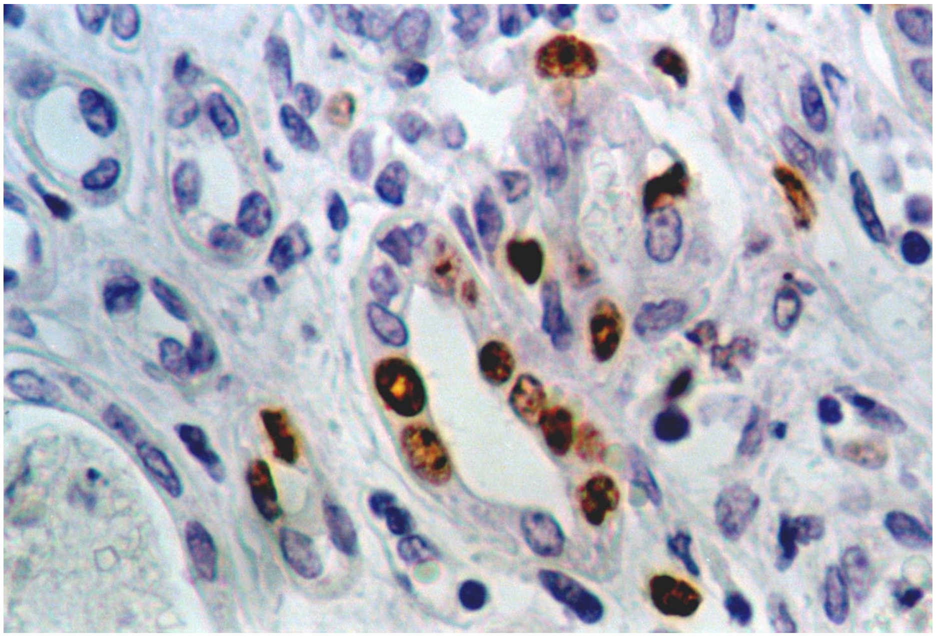

**FIGURE 7.** In high-power field, intense nuclear immunostaining is noted as well as cellular atypia.

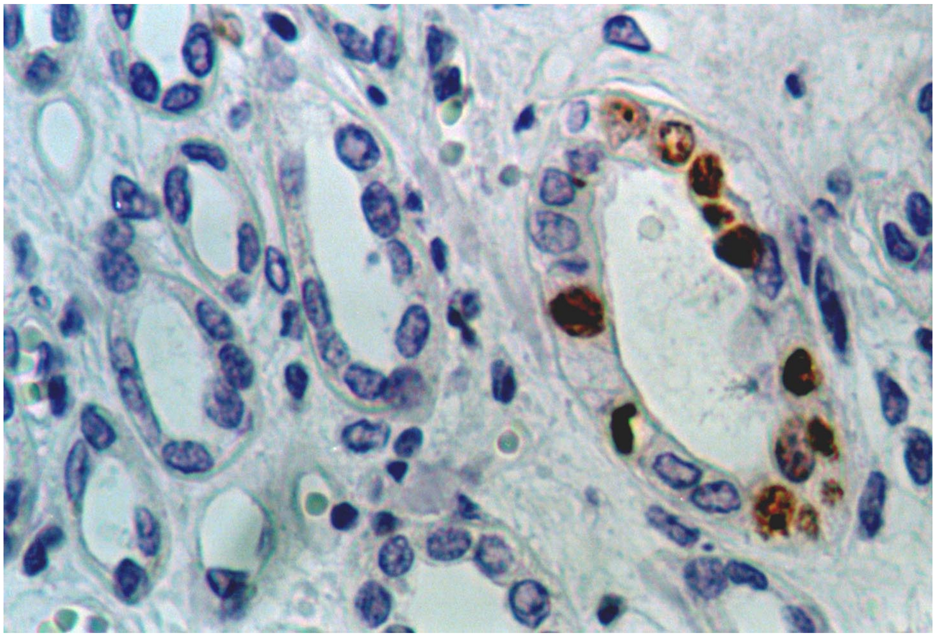

**FIGURE 8.** Another dysplastic tubule showing PCNA immunostaining and nuclear atypia.

## ADENOMA–CARCINOMA SEQUENCE

Epithelial hyperplasia is suggested to be a preneoplastic condition in polycystic kidney disease[28].

Preneoplastic lesions of the kidney in von Hippel-Lindau syndrome are well known[29,30]. Atypical cysts in von Hippel-Lindau syndrome show cytological and nuclear atypia with mitotical

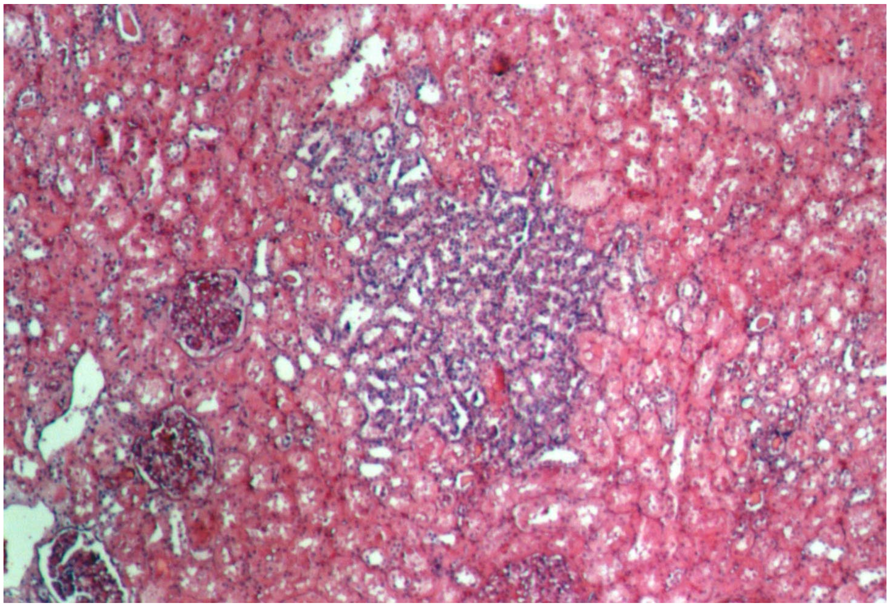

**FIGURE 9.** A small adenoma composed of closely packed tubules lined by regular, uniform cells.

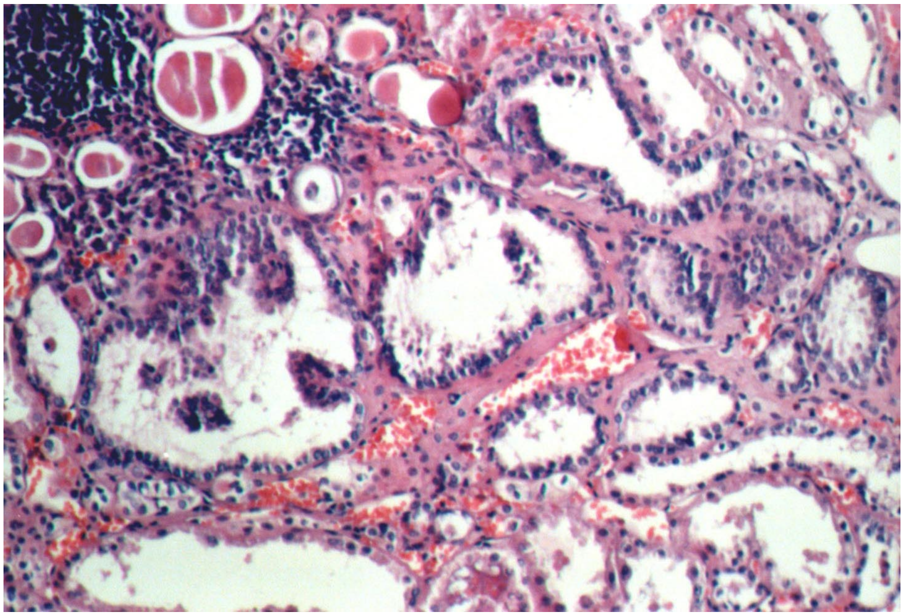

**FIGURE 10.** In a smaller adenoma, the cells are pleomorphic and crowded with a papillary architecture. Note the similar changes in the adjacent tubules.

activity and the RCCs developing in von Hippel-Lindau syndrome are presumed to progress from these atypical cysts[30].

Proximal tubule dysplasias and microneoplasms developing from these lesions were described in experimental cancer models[31]. RCC can be induced experimentally in male Syrian hamsters exposed to high dose estrogens and dysplastic changes has been described adjacent to the carcinoma[8,9]. In mice, most of the tumors induced by ethylnitrosourea were of papillary histopathological type and most of these tumors were associated with tubular dysplasia[31]. In human papillary RCC, adenoma-carcinoma sequence is a well-established feature[32,33] (Figs. 9,10).

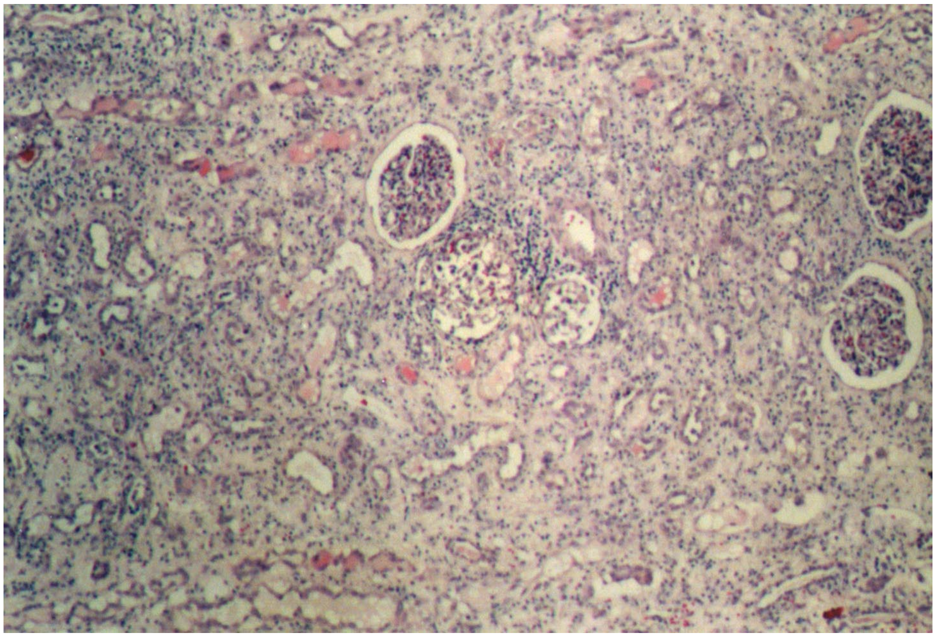

**FIGURE 11.** Carcinoma *in situ* foci in the periglomerular tubules in a collecting duct carcinoma case.

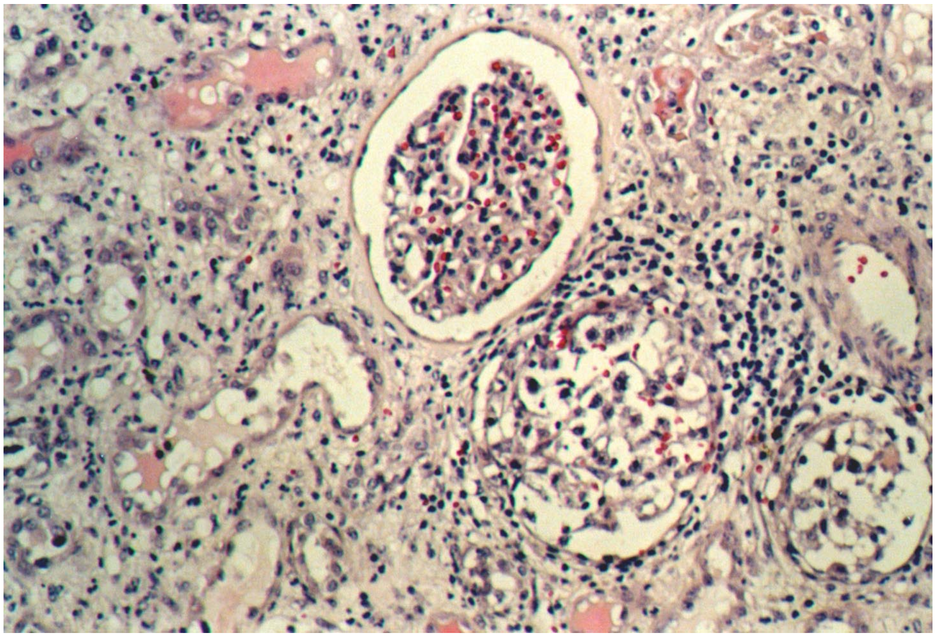

**FIGURE 12.** In the high power field, the lesion suggests to be a new tumor development or an extension of neoplastic cells along the tubular epithelium.

A well-known association between dysplasia and collecting duct carcinoma exists[11] (Figs. 11,12). Such an association in RCC of other subtypes is described in two recent series[34,35]. Collecting duct dysplasia was also reported in transplanted kidney in immunosupressed patients[36].

The association of oncocytic changes (i.e., diffuse oncocytic change in renal tubules, oncocytic or hybrid nodules [oncocytosis]**)** in chromophobe RCC and oncocytoma was described recently[13] (Figs. 13,14).

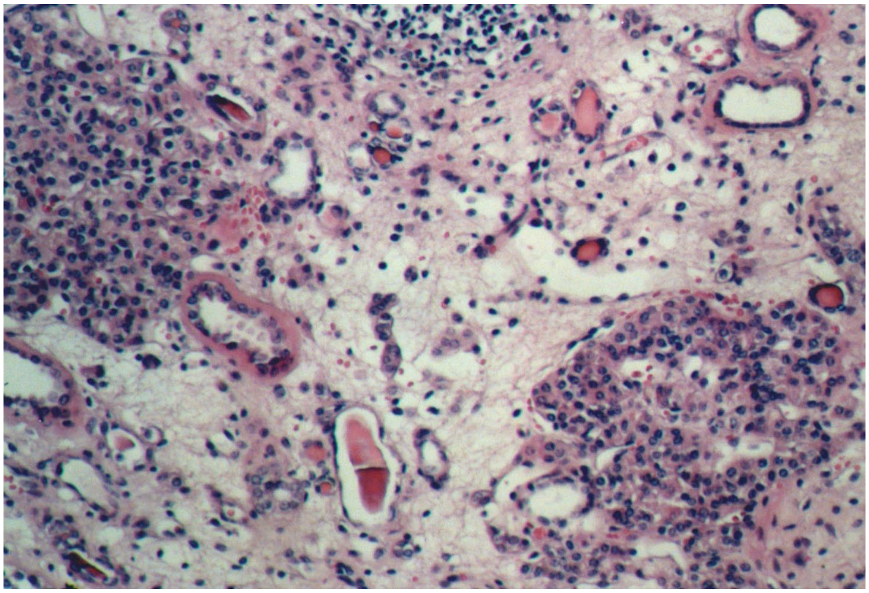

**FIGURE 13.** Two minute tumor foci of so-called “oncocytosis” in an oncocytoma case.

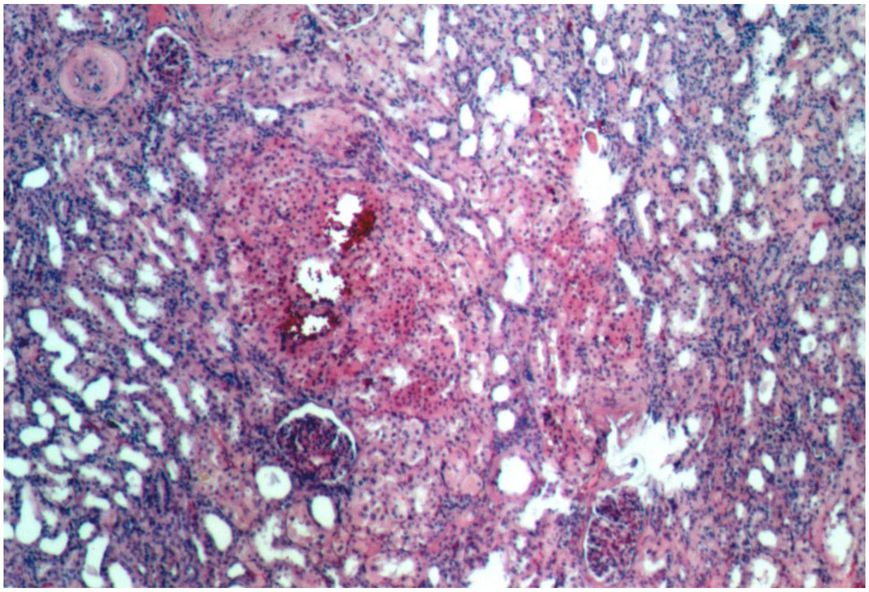

**FIGURE 14**. Two other minute tumor foci in a chromophobe RCC case, suggesting new tumor developments.

## TERMINOLOGY

The terminology of the premalignant lesions is well discussed for the uterine cervix. In that respect, there are some alternative schemes:

1. Dysplasia: mild, moderate, severe, and carcinoma *in situ*
2. Renal intratubular dysplasia I, II, and III
3. Renal intratubular neoplasia: low grade and high grade

Separating these lesions to three or four groups may lead to confusion and decreases the reproducibility[37,38]. The usage of a two-tier system seems to be more accurate and reproducible. By using the last system, severe dysplastic changes with known preneoplastic features can be separated from milder lesions with obscure preneoplastic capacity and biological behavior[19].

## CLINICAL IMPORTANCE AND MANAGEMENT

The incidence of RCC is increasing. The National Cancer Institute estimates 30,000 new cases and 12,000 deaths due to this disease in the U.S. There are no reliable tumor markers for RCC. Apart from imaging modalities, there is no method of early diagnosis for RCC today. Premalignant lesions of RCC like intratubular dysplasia can only be diagnosed by tissue sampling. Tissue sampling is not an easy decision in the kidney like other solid organs.

The risk of local recurrence after partial nephrectomy changes between 0.7 and 10% in different series[39,40,41,42]. In the series with a long follow-up, the incidence of contralateral disease was 6% with a 1% local recurrence incidence[42].

RCC may represent as multifocal tumors, and the risk of multifocality may rise to 25%[39]. The high multifocality rate and the lower risk of local recurrence contrasts. All the premalignant lesions do not need to progress to invasive carcinomas, and the spontaneous regression of RCC is a well-known event. Also, the follow-up periods in the series should not be overestimated. The progression rate may be slow not and may not manifest in the relatively short follow-up range of these studies.

## CONCLUSION

There are three, well-defined, distinct, precancerous lesions of the kidney—renal intratubular neoplasia, dysplastic changes within the cysts of the von Hippel-Lindau syndrome, and the adenoma-carcinoma sequence[19].

Epidemiological, pathological, and genetic evidence show that all of these lesions may proceed to invasive RCC. All invasive carcinomas of the kidney, however, do not need to develop from dysplastic lesions. It has also been shown that some RCC do not progress[35].

The fate of these lesions is not known. If these lesions are proved to be premalignant, then the role of nephron-sparing surgery in the treatment of localized small RCC should be redefined. The following question may be difficult to answer: "What should be the management if a renal biopsy performed for other medical problems reveals renal intratubular neoplasia?"

In two recent articles considering the recommendations for the reporting of renal tumors, renal intratubular neoplasia is not noted[43,44]. Interpretation and reporting of these lesions may yield further understanding and important resource for the biological nature of these lesions.
